# Fish burger enriched by olive oil industrial by‐product

**DOI:** 10.1002/fsn3.461

**Published:** 2017-05-27

**Authors:** Annamaria Cedola, Angela Cardinali, Matteo Alessandro Del Nobile, Amalia Conte

**Affiliations:** ^1^ Department of Agricultural Sciences Food and Environment University of Foggia Foggia Italy; ^2^ Institute of Sciences of Food Production, CNR Bari Italy

**Keywords:** antioxidant activity, dry olive paste, fish burger, phenolic compounds, sensory properties

## Abstract

Oil industry produces large volume of waste, which represents a disposal and a potential environmental pollution problem. Nevertheless, they are also promising sources of compounds that can be recovered and used as valuable substances. The aim of this work is to exploit solid olive by‐products, in particular dry olive paste flour (DOPF) coming from *Coratina* cultivar, to enrich fish burger and enhance the quality characteristics. In particular, the addition of olive by‐products leads to an increase of the phenolic content and the antioxidant activity; however, it also provokes a deterioration of sensory quality. Therefore, to balance quality and sensory characteristics of fish burgers, three subsequent phases have been carried out: first, the quality of DOPF in terms of phenolic compounds content and antioxidant activity has been assessed; afterward, DOPF has been properly added to fish burgers and, finally, the formulation of the enriched fish burgers has been optimized in order to improve the sensory quality. Results suggested that the enriched burgers with 10% DOPF showed considerable amounts of polyphenols and antioxidant activity, even though they are not very acceptable from the sensory point of view. Pre‐treating DOPF by hydration/extraction with milk, significantly improved the burger sensory quality by reducing the concentration of bitter components.

## 1 INTRODUCTION

Both solid and liquid waste resulting from the production, preparation and consumption of food industry are very large, thus posing increasing disposal and potential severe pollution problems. However, they are also promising sources of compounds that can be recovered and used as valuable substances (Schieber, Stintzing, & Carle, 2001). From the oil extraction process, regardless of the method used, extraction by pressure or centrifugation, wastewater and by‐products are formed in considerable amount. Specifically, the olive paste, in addition to the known applications as fuel and ingredient for making compost or as fertilizer for agricultural soil (Di Giovacchino, 2010), has recently attracted attention as natural source of bioactive compounds, mainly polyphenols, that generally contribute to the protective effect of the virgin olive oil (Cardinali et al., 2012; Scognamiglio et al., 2012). Olive paste is recognized as potential low‐cost starting material rich in phenolic compounds that could be extracted and applied as natural antioxidant to food and pharmaceutical applications. The main phenolic compounds identified in olive waste are: hydroxytyrosol, tyrosol, caffeic acid, p‐coumaric acid, catechol, 4‐methylcatechol, p‐hydroxybenzoic acid, vanillic acid, syringic acid, gallic acid, catechin, apigenin, kaempferol, luteolin, quercetin, cyanidin, peonidin, nuzhenide, ligstroside, verbascoside, caffeoyl‐6‐secologanoside, comselogoside, and some polymeric compounds (D'Antuono et al., 2014; De Marco, Savarese, Paduano, & Sacchi, 2007; El‐Abbassi, Kiai, & Hafidi, 2012; Obied, Prenzler, & Robards, 2008). Epidemiological data highlight the olive compounds role in the prevention of different diseases. The beneficial effects seem to be attributable to its secondary metabolites content. In fact, phenolic alcohols (Bianco et al., 2006; Bianco, 2000), flavonoids (Altiok, Baycin, Bayraktar, & Ulku, 2008), phenylethanoid glycosides (Amiot, Fleuriet, & Macheix, 1986), secoiridoids (Karioti et al., 2006), and tannins (Ryan, Antolovich, Prenzler, Robards, & Lavee, 2002), all found at high concentration in olive oil waste, appear to be capable of inducing anti‐carcinogenic effects in large intestinal cancer cell models, in animals and humans.

Many researches have shown that by‐products generally exert an high nutritional value, thus suggesting their utilization as food ingredients and provide a valid solution for pollution problems connected with food processing (Lario et al., 2004). Calvo, García, and Selgas (2008) produced meat sausages enriched with lycopene by means of direct addition of tomato skins, previously dried. Padalino et al. (2015) incorporated tomato peels in durum wheat spaghetti to increase the nutritional quality without affecting the sensory quality. Fernández‐López, Sendra, Sayas‐Barberá, Navarro, and Pérez‐Alvarez (2008) used orange peel in dry fermented sausage, thus giving a product with high level of phenolic compounds, without compromising the general flavor. In order to realize novel food with by‐products is necessary to consider if their incorporation alters the sensory properties, thus suggesting careful selection of the type and the amount of ingredients to be added and proper technological options to be adopted (Bonet, Blaszczak, & Rosell, 2006; Lodi, Tiziani, & Vodovatz, 2007). Many studies have already been performed to extract bioactive compounds from olive pomace by using solvents such as methanol, ethanol, or ethyl acetate (Fernández‐Bolaños, Rodríguez, Rodríguez, Guillén, & Jiménez, 2006). Until now, no direct addition of waste oil industry to food has been realized because these types of by‐products have very bitter and spicy taste. Therefore, the main aim of this work was to develop a novel fish burger enriched with innovative dried olive pomace, rich in phenolic compounds, to increase the nutritional quality, the health promoting principles like antioxidant and other beneficial phytochemicals. To reduce the bitter taste and to obtain an acceptable final product, dried olive pomace flour has been properly pre‐treated prior to the addition to the burger.

## 2 MATERIALS AND METHODS

### 2.1 Raw materials

The olive paste was obtained by a local olive mill (Bisceglie, Bari, Italy) from the organic *Coratina* cultivar milled using a Pieralisi Leopard with DMF technology (Multi Phases Decanter). Leopard is the two‐phase decanter that can combine modern extraction technology without the addition of water. It produces a dehydrated husk similar to the one coming from a three phases decanter; it also recovers a certain quantity of husk (olive pasta, OP) made up of wet pulp without any traces of kernel. The olive paste was dried at 35°C in a dryer (SG600, Namad, Rome, Italy) for 72 hr. The dried olive paste was reduced in a fine powder (<500 μm) by a hammer mill (16/BV‐Beccaria s.r.l., Cuneo, Italy) and then stored at 4°C until further utilization. The fish (tuna trance) was purchased from a local farm, Minaba srl (Manfredonia, Foggia, Italy).

### 2.2 Fish burger preparation

Fish burger (40 g) were manufactured using tuna trance, previously thawed and chopped until to a diameter of about 12 mm (28 g), mixed with potato flour (2 g), parsley (0.2 g), salt (0.2 g), and a whey protein based crumb soaked in extra‐virgin oil, as reported by Del Nobile et al. (2009). The mixture was added with 10% w/w of dried olive pomace flour (DOPF). DOPF was added to the burger formulation with and without any pre‐treatment. In particular, two different pre‐treatments were adopted: hydration and hydration/extraction. In the first case, DOPF was hydrated either with water or milk in a DOPF/liquid ratio of 1:1 for 1 hr, referred as DOPF‐H. In the second case, DOPF was first hydrated with either water or milk in a DOPF/liquid ratio of 1:5 for 1 hr (hydration stage) and then, the excess liquid was drained (separation stage), referred as DOPF‐H/E. The control sample was prepared without the olive paste. The dough was mixed for a few minutes and then the burgers were manually realized (diameter 5 cm and height 1 cm). Finally, the burgers were cooked in an electric convention oven (H2810, Hugin, Milan, Italy) at 240°C for 12 min.

### 2.3 Chemicals

Folin‐Ciocalteu reagent, gallic acid monohydrate, Trolox (6‐hydroxy‐2,5,7,8‐tetramethylchroman‐2‐carboxylic acid), potassium persulfate (K_2_S_2_O_8_), ABTS (2,2‐azino‐bis(3‐ethylbenzothiazoline‐6‐sulfonic acid diammonium salt), 2,2‐Diphenyl‐1‐picrylhydrazyl (DPPH radical), aluminum chloride (AlCl_3_), sodium hydroxide solution (NaOH), sodium nitrite (NaNO_2_), and quercetin were supplied from Sigma‐Aldrich (Milan, Italy). Anhydrous sodium carbonate (Na_2_CO_3_) was supplied from Carlo Erba (Milan, Italy). For the preparation of the phosphate buffered saline (PBS), the following salts were used: sodium phosphate monobasic monohydrate (H_2_NaO_4_P·H_2_O) and sodium phosphate dibasic heptahydrate (HNa_2_O_4_P·7H_2_O). These were purchased from Sigma‐Aldrich (Milan, Italy). All reagents were of analytical grade.

### 2.4 Total phenols, flavonoids, and antioxidant activity determination

To determine total phenols, flavonoids, and antioxidant activity, the extraction was performed as described by Meneses, Martins, Teixeira, and Mussatto (2013) with slight modifications. Burger with and without olive paste, both raw and cooked, were dried at 35°C with a ventilated stove (BINDER GmbH, Tuttlingen, Germany), than milled to obtain a powder. For the extraction, 1 g of dried sample was mixed with 20 ml of equal mixture water: ethanol (v/v) in Erlenmeyer flasks, which were duly covered to avoid solvent loss, and maintained during 30 min in a water‐bath (GRANT OLS200, Cambridge, England) with magnetic agitation at 60°C. After this time, the extracts were filtered with Nylon 0.45 μm to obtain a clear supernatant. The volume of extract recovered after each extraction was quantified and used for calculation. Triplicate extractions were made for each sample.

### 2.5 Chemical analyses

#### 2.5.1 Determination of total phenolic compounds

Total phenolic compounds were determined by UV‐vis spectrophotometry according to Folin‐Ciocalteu method (Spinelli, Conte, Lecce, Incoronato, & Del Nobile, 2015). In particular, DOPF was 1:20 diluted with water before analysis, while the extracts obtained from samples, previously described, were analyzed without any dilution. Specifically, 0.5 ml of dry olive pasta or burger extract was mixed with 2.5 ml of Folin‐Ciocalteu reagent (diluted 1:10 with water) and, after 5 min, 2 ml of Na_2_CO_3_ (75 g/L) was added. The sample was kept in darkness at room temperature for 2 hr. The equal solution water: ethanol (v/v) was used as control sample. The absorbance was measured at 740 nm by an UV‐vis spectrophotometer (UV1800, Shimadzu Italia s.r.l., Milan, Italy). Total phenolic compounds were quantified by a calibration curve previously built (3.12–100 mg/L; *R* ^2^ = 0.9989) using standard solution of gallic acid; the total phenolic content was expressed as mg gallic acid/g of dry weight (dw). All tests were carried out in triplicate.

#### 2.5.2 Determination of total flavonoids

Total flavonoids content both in DOPF and in all the burger's extracts was determined by aluminum chloride colorimetric method, according to Chiung‐Tsu, Tzu‐Hao, Tai‐Hao, Frang‐Yi, and Hui‐Yin (2015) with some modifications, using quercetin as standard. Extracts (0.5 ml) prepared as previously described, were mixed with 2 ml of distilled water and 150 μl of a 5% sodium nitrite (NaNO_2_) solution. After 6 min, 150 μl of a 10% aluminum chloride (AlCl_3_) solution was added and the mixture was allowed to stand for 6 min. Finally, 1 ml of 1mol/L sodium hydroxide (NaOH) was added until had volume was made up to 5 ml with distilled water. Then, the solutions were mixed and for each sample the absorbance was read in triplicate against blank at 415 nm. The standard curve was prepared using quercetin as standard in the range 12.5–200 mg/L (*R* ^2^ = 0.9954) and total amount of flavonoids was expressed in mg of quercetin/g of dry weight (dw).

#### 2.5.3 Trolox equivalent antioxidant capacity assay

The antioxidant activity was evaluated using ABTS assay according to the method of Arnàiz et al. (2016), with slight modifications. The ABTS assay is based on the ability of antioxidants to interact with the radical cation 2,2‐azino‐bis (3‐ethylbenzothiazoline‐6‐sulfonic acid) inhibiting its absorption at 728 nm. 7 mmol/L ABTS stock solution and 140 mmol/L potassium persulfate were utilized. The ABTS radical cation (ABTS^∙+^) was obtained by reacting ABTS stock solution with 2.45 mmol/L potassium persulfate (final concentration) and leaving the mixture in the dark at room temperature for 12–16 hr. The ABTS^∙+^ solution was diluted to an absorbance 0.700 ± 0.020 at 728 nm, with 5 mmol/L phosphate buffered saline (pH 7.4). Then, 300 μl of sample extract was added to 2.2 ml of ABTS^∙+^ diluted solution and after 6 min at room temperature the mixture was measured through a spectrophotometer (UV1800, Shimadzu Italia s.r.l., Milan, Italy) at 728 nm. A calibration curve was previously built using 6‐hydroxy‐2,5,7,8‐tetramethylchroman‐2‐carboxylic acid (Trolox) as standard, at concentrations between 0.94 and 40 mg/L (*R* ^2^ = 0.9995) and the antioxidant activity was expressed as mg Trolox equivalents for gram of dry weight (dw). The analyses were carried out in triplicate.

#### 2.5.4 Scavenging effects on DPPH radicals

The free radical activity was defined by measuring the ability of the extracts to scavenge the free radical 2,2‐diphenyl‐1‐picrylhydrazyl (DPPH). The antioxidant activity was determined using a method described by Meneses et al. (2013) with some modifications. For the reactions, 100 μl of each extract were added to 2.9 ml of DPPH solution (6 × 10^−5^ mol/L in ethanol). The resulting solutions were vortexed and allowed to stand for 30 min in darkness at room temperature. Then the absorbance was measured at 517 nm in a spectrophotometer (UV1800, Shimadzu Italia s.r.l., Milan, Italy), using ethanol as blank. The control solution was constituted by ethanol instead of the sample. The radical scavenging activity was expressed as the inhibition percentage using the Equation (1), where A_C_ and A_S_ are the absorbance of the control solution and the absorbance of the sample solution respectively.

(1) $$\%\;\text{Inhibition of DPPH}=(1-A_{S}/A_{C})\times 100$$

### 2.6 Sensory analysis

Fish burger samples were submitted to a panel of eight trained tasters in order to evaluate the sensory attributes. The panelists were selected on the basis of their sensory skills (ability to accurately determine and communicate the sensory attributes as appearance, odor, flavor, and texture). They were asked to indicate color, odor, and texture of raw burgers. Color, odor, taste, texture, juiciness, and tenderness were evaluated on cooked fish burgers. To the aim, a nine‐point scale, where 1 corresponded to extremely unpleasant, 9 to extremely pleasant and 5 to the threshold acceptability, was used to quantify each attribute. On the basis of the afore‐mentioned attributes, panelists were also asked to score the *overall quality* of both cooked and uncooked products using the same nine‐point scale.

### 2.7 Statistical analysis

Experimental data were compared by a one‐way analysis of variance (ANOVA). Duncan's multiple range test, with the option of homogeneous groups (*p* < .05), was carried out to determine significant differences between samples. STATISTICA 7.1 for Windows (Stat Soft, Inc., Tulsa, OK, USA) was used.

## 3 RESULTS AND DISCUSSION

The work has been organized in three subsequent phases: first, the dry olive paste flour (DOPF) has been produced and characterized for total polyphenols and flavonoids content and antioxidant capacity; afterward DOPF has been added to fish burgers to enhance the nutritional quality; finally, the enriched fish burgers were optimized in order to also improve the sensory quality.

### 3.1 Production and characterization of dry olive paste

As reported above, to render olive paste shelf stable, it was dried by a low temperature drying cycle to preserve olive paste quality characteristics. The total phenolic compounds (mg gallic acid/g dw), the flavonoids (mg quercitin/g dw), and the antioxidant activity (mg Trolox/g dw) of DOPF were shown in Table 1. The obtained results indicate that the dry olive paste has a high phenols content, equal to 31.16 mg acid gallic/g dw, not much lower than that reported by Chiung‐Tsu et al. (2015), who studied the total phenolic compounds and antioxidant properties of Chinese olive fruit. Also flavonoids, that constitute the largest group of plant phenolic accounting for over half of the eight thousand naturally occurring phenolic compounds (Asfaw & Demissew, 1994), were present in high amounts in dry olive paste (61.24 mg quercetin/g dw). Results confirm that also the antioxidant activity of DOPF recorded by both methods, ABTS assay and DPPH radical scavenging method, was very high. In particular, the dry olive paste present 43.33 mg Trolox/g dw as result of ABTS assay and 88.19% as sequestrating activity on DPPH, thus suggesting that its addition to food could greatly contribute to enhance the nutritional quality. It is also worth noting that according to Alonso, Guillen, Barroso, Puertas, and Garcia (2002) there was a positive correlation between the antioxidant activity and the total polyphenol content.

**Table 1:** Total phenols, total flavonoids and antioxidant activity of dry olive paste flour (DOPF) and burgers with and without the addition of DOPF

Sample	Total phenols (mg GAE/g dw) ± SD	Total flavonoids (mg QE/g dw) ± SD	Antioxidant activity (mg Trolox/g dw) ± SD	Antioxidant activity DPPH(%) ± SD
DOPF	31.16 ± 0.29^a^	61.24 ± 0.89^a^	43.33 ± 0.01^a^	88.19 ± 0.00^a^
R‐CNT	0.47 ± 0.01^b^	0.03 ± 0.03^b^	0.59 ± 0.02^b^	1.12 ± 0.00^b^
C‐CNT	0.51 ± 0.01^b^	0.05 ± 0.03^b^	0.61 ± 0.01^b^	1.37 ± 0.00^c^
R‐BURGER‐DOPF	6.15 ± 0.18^c^	9.97 ± 0.50^c^	6.06 ± 0.02^c^	85.77 ± 0.00^d^
C‐BURGER‐DOPF	6.55 ± 0.18^d^	10.51 ± 0.32^c^	5.99 ± 0.01^d^	84.87 ± 0.00^e^

GAE, gallic acid equivalents; QE, quercetin equivalent; DPPH, 2,2‐diphenyl‐1‐picrylhydrazyl. The different letters show significant difference between means of triplicate determinations (*p *<* *.05). R‐CNT, uncooked burger without DOPF; C‐CNT, cooked burger without DOPF; R‐BURGER‐DOPF, uncooked burger with DOPF; C‐BURGER‐DOPF, cooked burger with DOPF.

### 3.2 Dry olive paste enriched burger

#### 3.2.1 Nutritional quality

The total phenolic content, the flavonoids and the antioxidant activity of fish burgers enriched with 10% DOPF were determined on both raw and cooked products and presented in Table 1. The content of total phenols in the experimental samples, expressed as gallic acid equivalents (GAEs), varied from 0.47 mg GAE/g dw in the CNT sample to 6.15 mg GAE/g dw in the enriched burger, which represent a very big increase. It is worth noting that the cooking process slightly increased the phenols amounts (6.55 mg GAE/g dw) most probably due to the fact that in this step the bounded compounds become available (Marinelli, Padalino, Nardiello, Del Nobile, & Conte, 2015). Abdel‐Aal and Rabalski (2013) stated that the effects of cooking on polyphenols are not always the same; they depend on the type of bioactive compound involved and on the type of food product. Baking for example, in some cases is reported to slightly increase the phenols content (Gelinas & McKinnon, 2006), whilst others have claimed that phenolic compounds are destroyed during baking (Leenhardt et al., 2006). Similarly to phenols, also the flavonoids content of DOPF enriched burgers, both raw and cooked, is much higher than the corresponding CNT sample (9.97 to 0.03 mg QE/g dw). Flavonoids as all phenolics compounds exhibit a variety of activities, including anti‐inflammatory, antioxidant, and antiallergenic activity and also reduce the risk of cardiovascular disease and cancer (Srivastava & Gupta, 2007). According to the recorded data, dry olive paste enrichment leads to a significant increase in burger antioxidant capacity. In particular, the active samples show higher antioxidant activity than the CNT, 0.59 against 6.06 mg Trolox/g for the ABTS method, and 1.12 against 84.87% for the DPPH method. This antioxidant activity turned out to be in agreement with the total phenolic content previously reported. As a fact, the DPPH method is based on the ability of DPPH radical to react with hydrogen donor species, such as phenols and flavonoids, present in the extract material (Brand‐Williams, Cuvelier, & Berset, 1995). According to Shabir et al. (2011), the correlation between the DPPH results and the total phenols and flavonoids content in the extracts was positive for both cases, indicating that the antioxidant activity increased with the increasing of the total phenols and flavonoids concentration.

#### 3.2.2 Sensory quality

The sensory properties of the investigated samples were evaluated by eight trained panelists. The results are listed in Table 2 for the burgers with and without the addition of dry olive paste (uncooked and cooked). As far as the sensory quality of raw burgers is concerned, the addition of DOPF in the fish burgers determined a worsening of the color (4.58) due to the dark green color of the olive paste. The same trend was also found in terms of texture, with a score (5.08) lower than the control sample (7.58). In particular, the addition of dry olive flour gave burgers more bulky and less juicy (Spinelli et al., 2015). So, the *overall quality* was generally compromised and reached score under the acceptability threshold.

**Table 2:** Sensory characteristics of uncooked and cooked burger samples

Sample	Uncooked sample				Cooked sample						
	Color	Odor	Texture	Overall Quality	Color	Odor	Taste	Texture	Juiciness	Tenderness	Overall Quality
**CNT**	7.58 ± 0.38^b^	7.83 ± 0.26^b^	7.58 ± 0.49^b^	7.50 ± 0.32^b^	6.58 ± 0.38^a^	7.50 ± 0.45^b^	7.58 ± 0.20^b^	7.08 ± 0.49^b^	6.67 ± 0.61^b^	7.42 ± 0.38^b^	6.92 ± 0.38^b^
**BURGER‐ DOPF**	4.58 ± 0.49^a^	6.58 ± 0.49^a^	5.08 ± 0.38^a^	4.83 ± 0.52^a^	5.83 ± 0.52^a^	4.50 ± 0.63^a^	3.17 ± 0.52^a^	5.50 ± 0.45^a^	5.25 ± 0.61^a^	6.33 ± 0.41^a^	3.33 ± 0.61^a^

^a–b^Data in columns with different superscripts are significantly different (*p* < .05).

CNT, burger without DOPF; BURGER‐DOPF, burger with DOPF.

As regard cooked burgers, both color and texture scores (5.83 and 5.50, respectively) recorded values lower than the control (6.58 and 7.08), but, odor and taste of enriched burgers (4.50 and 3.17, respectively) were the main responsible attributes for product unacceptability (score = 3.33). The enriched burger had very bitter and spicy taste, probably due of the high content of polyphenols, in particular oleuropein, the characteristic compound of olive oil, responsible for the characteristic bitter taste (Cardinali et al., 2010). On the basis of the recorded sensory characteristics, further efforts were aimed to improve the sensory quality of the enriched burgers.

### 3.3 Optimization of DOPF enriched fish burgers

The experimental findings recorded in the previous step highlight that bitter and spicy taste is the main problem that limits the use of waste oil industry as food ingredient. Therefore, to face the problem, DOPF was pre‐treated with either water or milk (DOPF‐H and DOPF‐H/E) prior to be added to the burger formulation. The new fish burger sensory properties are listed in Table 3. As can be seen, a simple hydration of DOPF with either water or milk, does not contribute to improve the sensory quality. In particular, the cooked DOPF‐H‐H_2_O and DOPF‐H‐MILK samples recorded low values of odor and taste, thus receiving a global quality score under the acceptability threshold (3.83 and 4.33). Conversely, the hydration/extraction with either water or milk significantly improved the sensory quality. As can be inferred from the table, the *overall quality* of DOPF‐H/E‐H_2_O (6.67) and DOPF‐H/E‐MILK (7.17) samples recorded a score very similar to the CNT (7.50) sample, for both cooked and uncooked burger. In fact, the hydration/extraction with either water or milk leads to a substantial decrease of the bitter taste related to DOPF. In particular, the bitterness of cooked sample markedly decreased when DOPF‐H/E‐MILK is used, probably due to the fact that drained milk retains part of bitter substances such as polyphenols. This is in agreement with Pripp, Busch, and Vreeker (2004), who observed that the presence of milk proteins, sodium caseinate, in emulsion with the extra virgin olive oil attenuates the intensity of bitterness perception. They indicated that the presence in the oil of an amount comprised between 1 and 4% of caseinate, results in a reduction of bitter perception by about 60%. The formation of protein‐phenols ties would prevent the interaction between the salivary proteins and the taste receptors to interact with the phenolic molecules, reducing the perception of bitterness (Pripp et al., 2004). These results are also confirmed by the analysis made on the drained liquid, either water or milk (data not shown); data highlighted that the compounds responsible for the bitter taste passed in the extracting liquid, thus reducing their concentration in the burger.

**Table 3:** Sensory characteristics of uncooked and cooked burger samples prepared in the last step

Sample	Uncooked sample				Cooked sample						
	Color	Odor	Texture	Overall quality	Color	Odor	Taste	Texture	Juiciness	Tenderness	Overall quality
CNT	7.58 ± 0.38^c^	7.83 ± 0.26^b^	7.58 ± 0.49^b^	7.50 ± 0.32^b^	6.58 ± 0.38^a^	7.50 ± 0.45^a^	7.58 ± 0.20^c^	7.08 ± 0.49^a^	6.67 ± 0.61^a^	7.42 ± 0.38^a^	7.50 ± 0.45^a^
DOPF H‐H_2_O	5.00 ± 0.63^a^	6.92 ± 0.38^a^	6.58 ± 0.27^a^	6.83 ± 0.52^a^	6.75 ± 0.52^a^	5.42 ± 0.49^b^	4.00 ± 0.71^a^	5.83 ± 0.52^b^	5.67 ± 0.52^b^	6.83 ± 0.75^a^	3.83 ± 0.68^b^
DOPF H‐MILK	4.83 ± 0.26^a^	6.67 ± 0.41^a^	6.50 ± 0.32^a^	5.17 ± 0.41^a^	6.92 ± 0.38^a^	6.00 ± 0.45^b^ ^,c^	4.33 ± 0.26^a^	6.08 ± 0.18^b^	5.92 ± 0.20^b^	7.08 ± 0.38^a^	4.33 ± 0.26^b^
DOPF H/E‐H_2_O	6.58 ± 0.38^b^	7.33 ± 0.41^a^^,^^b^	7.25 ± 0.27^b^	6.86 ± 0.38^b^	7.00 ± 0.32^a^	6.67 ± 0.52^a^ ^,c^	6.75 ± 0.42^b^	6.83 ± 0.52^a^	6.75 ± 0.52^a^	7.42 ± 0.20^a^	6.67 ± 0.41^a^
DOPF H/E‐MILK	6.67 ± 0.52^b^	7.25 ± 0.52^a^^,^^b^	7.25 ± 0.27^b^	6.83 ± 0.52^b^	7.33 ± 0.52^a^	7.42 ± 0.38^a^	7.08 ± 0.38^b^ ^,c^	7.42 ± 0.38^a^	7.42 ± 0.38^a^	7.42 ± 0.38^a^	7.17 ± 0.26^a^

^a–c^Data in columns with different superscripts are significantly different (*p* < .05).

DOPF H‐H_2_O, burger with DOPF hydrated with water; DOPF H‐MILK, burger with DOPF hydrated with milk; DOPF H/E‐H_2_O, burger with DOPF after hydration/extraction with water; DOPF H/E‐MILK, burger with DOPF after hydration/extraction with milk.

As one would expect, although the hydration/extraction of DOPF improved the sensory properties, reduced polyphenols amount and antioxidant activity were recorded in these last samples (Table 4). In particular, the hydration/extraction process with milk leads to a greater loss of bioactive compounds. The total phenols varied from 6.47 mg GAE/g dw to 1.86 mg GAE/g dw that in any case is 3.64 times higher than the control sample. Similar results were also found by Ye, Fan, Xu, and Liang (2013), who studied the interactions between polyphenols and milk proteins. They reported that casein micelles are able to bind highly polymerized polyphenols. In the case of hydration/extraction process with water, a lower loss of polyphenols was observed. The polyphenols content decreased from 6.13 mg GAE/g dw to 4.13 mg GAE/g dw that is eight times greater than the control. Regarding the antioxidant activity, DOPF‐H/E samples exerted lower value than the DOPF‐H samples, as a consequence of the extraction process (Liu, Zhao, Gan, & Ni, 2015).

**Table 4:** Chemical data of burger samples prepared in the last step

Sample	Total phenols (mg GAE/g dw) ± SD	Total flavonoids (mg QE/g dw) ± SD	Antioxidant activity (mg Trolox/g dw) ± SD	Antioxidant activity DPPH(%) ± SD
R‐CNT	0.47 ± 0.01^a^	0.03 ± 0.03^a^	0.59 ± 0.02^b^	1.12 ± 0.00^a^
C‐CNT	0.51 ± 0.01^a^	0.05 ± 0.03^a^	0.61 ± 0.01^b^	1.37 ± 0.00^b^
R‐DOPF‐H‐H_2_O	6.08 ± 0.18^b^	8.86 ± 0.29^c^	6.20 ± 0.01^g^	85.53 ± 0.00^i^
C‐DOPF‐H‐H_2_O	6.13 ± 0.17^b^	9.26 ± 0.55^c^	5.77 ± 0.01^a^	85.73 ± 0.00^l^
R‐DOPF‐H‐MILK	5.66 ± 0.08^g^	8.30 ± 0.45^f^	5.79 ± 0.01^a^	82.45 ± 0.01^h^
C‐DOPF‐H‐MILK	6.47 ± 0.05^h^	11.45 ± 0.39^g^	5.78 ± 0.02^a^	86.94 ± 0.01^m^
R‐DOPF‐H/E‐H_2_O	3.65 ± 0.17^e^	3.42 ± 0.23^d^	3.39 ± 0.01^e^	57.97 ± 0.01^f^
C‐DOPF‐H/E‐H_2_O	4.13 ± 0.30^f^	5.77 ± 0.51^e^	3.50 ± 0.01^f^	73.22 ± 0.02^g^
R‐DOPF‐H/E‐MILK	1.61 ± 0.07^c^	1.07 ± 0.07^b^	1.63 ± 0.01^c^	32.46 ± 0.0^c^
C‐DOPF‐H/E‐MILK	1.86 ± 0.07^d^	1.33 ± 0.04^b^	2.26 ± 0.01^d^	46.21 ± 0.00^d^

^a–m^Data in columns with different superscripts are significantly different (*p* < .05).

R‐ and C‐DOPF‐H‐H_2_O: uncooked and cooked burger with DOPF hydrated with water; R‐ and C‐DOPF‐H‐MILK: uncooked and cooked burger with DOPF hydrated with milk; R‐ and C‐DOPF‐H/E‐H_2_O: uncooked and cooked burger with DOPF after hydration/extraction with water; R‐and C‐DOPF‐H/E‐MILK: uncooked and cooked burger with DOPF after hydration/ extraction with milk.

## 4 CONCLUSION

In this study, the impact of DOPF addition, an olive oil industry by‐product, on both sensory and quality characteristics of fish burgers was evaluated. In the first experimental phase, DOPF was characterized for total bioactive composition. The obtained results indicate that DOPF has a high content of phenols, flavonoids and consequently a high antioxidant activity, capable to improve the nutritional characteristics of food. Therefore, DOPF was added to fish burgers at concentration of 10% to enhance the nutritional quality. As expected, the DOPF enrichment considerably improved the fish burger quality, but it negatively influenced the sensory properties due to a very bitter and spicy taste that became the products unacceptable. To reduce the bitter defect, DOPF was pretreated with either water or milk before it was added to burger's formulation. Between the various pretreatments, milk hydration/extraction significantly improved the burger sensory quality by reducing the polyphenols concentrations and consequently the characteristic bitter taste and spicy note. To sum up, the polyphenolic enrichment of almost 8 times compared to the control with the preliminary treatment of DOPF to face the sensory bitter taste, permits to consider the new fish burger suitable for a balanced diet with the possibility to be regarded as a new functional food.

## CONFLICT OF INTEREST

The authors report no conflicts of interest.
